# Day-of-the-week variation of sudden unexpected cardiac death^☆^

**DOI:** 10.1016/j.ijcrp.2024.200246

**Published:** 2024-02-09

**Authors:** Hanna Latola, Severi M. Mattila, Lasse Pakanen, Lauri Holmström, Janna Kauppila, Olavi H. Ukkola, M Juhani Junttila, Heikki V. Huikuri, Juha S. Perkiömäki

**Affiliations:** 1 Research Unit of Biomedicine and Internal Medicine, Medical Research Center Oulu, University of Oulu and Oulu University Hospital, Oulu, Finland; 2 Forensic Medicine Unit, Finnish Institute for Health and Welfare, Oulu, Finland; 3 Department of Forensic Medicine, Research Unit of Biomedicine and Internal Medicine, Medical Research Center Oulu, University of Oulu, Oulu, Finland

**Keywords:** Sudden cardiac death, Septadian variation, Medico-legal autopsy

## Abstract

**Background:**

There has been some controversy about the day-of-the-week (septadian) variation of unexpected sudden cardiac death (SCD).

**Methods:**

We evaluated the incidence of unexpected SCD on different days of the week in a consecutive series of 5869 SCD victims from Northern Finland [the FINGESTURE study (Finnish Genetic Study of Arrhythmic Events)]. As it is mandatory in Finland, a medico-legal autopsy was performed on all unexpected sudden death victims. The autopsies were performed between the years 1998–2017.

**Results:**

The mean incidence of unexpected SCD was higher at weekends (during the days from Friday to Sunday, peaking on Saturday) than during the days from Monday to Thursday (8.54 ± 0.72 vs. 7.22 ± 0.19 SCDs per day of the week per 100,000 inhabitants per year, p < 0.001). Regardless of sex or ischemic versus non-ischemic etiology of SCD, the distribution of the occurrence of SCD among the days of the week was similar compared with the whole SCD cohort.

**Conclusion:**

The incidence of unexpected SCD was highest at weekends (during the days from Friday to Sunday, peaking on Saturday).

## 1 Introduction

Sudden cardiac death (SCD) is a significant mode of death worldwide, causing from 10 to 20% of all deaths [1]. SCD is the most common cause of cardiovascular mortality accounting for approximately 50% of deaths due to cardiovascular diseases. Different studies have looked into day-of-the-week (septadian) variations in the incidence of cardiac events and SCD. The results have been variable. Some previous studies have found that there is a higher incidence of SCD on Mondays [[2], [3], [4]]. A possible explanation for this has been proposed to be stress-induced sympathetic nervous system activation associated with early wake-up and return to work on Mondays that could predispose the most vulnerable subjects to SCD. However, more recent studies have suggested that there is no peak in sudden cardiac arrest on Mondays [5] and that there might recently have been a loss of septadian variation in sudden cardiac arrest [6]. As there has been some controversy about the septadian variation of unexpected SCD, we evaluated whether there was day-of-the-week variation in the occurrence of SCD in the consecutive series of 5869 unexpected SCD victims. As it is mandatory in Finland, a medico-legal autopsy was performed on all unexpected sudden death victims.

## 2 Methods

According to Finnish law, a medico-legal cause-of-death investigation is performed if the death is not known to be caused by a disease, the deceased has not been treated during his/her last disease by a doctor, the death is or is suspected to be caused by non-natural causes, or the death is otherwise unexpected.

The data for this study is from the Finnish Genetic Study of Arrhythmic Events (FINGESTURE). The study population consists of autopsies on unexpected out-of-hospital SCD victims from the defined geographical area of the Oulu University Hospital district in northern Finland between the years 1998 and 2017. Autopsy-confirmed SCD victims (n = 5869) were gathered from this population with a wide range of data on the deceased from medical records and autopsy and toxicological findings, including blood alcohol levels, and from interviews with next of kin. Blood alcohol levels were measured in 3795 SCD victims, i.e., whenever there was any suspicion of alcohol use prior to SCD. All unexpected sudden death victims underwent a medico-legal autopsy as obligated by Finnish law. The autopsies were performed in the Finnish Institute for Health and Welfare, Oulu, Finland, or at the Department of Forensic Medicine, University of Oulu, Oulu, Finland by experienced forensic pathologists using a standardized protocol. Non-cardiac causes of sudden death, such as cerebrovascular events, aortic rupture, pulmonary embolism, trauma, and intoxication, were excluded from the series. The cohort was subcategorized into ischemic or non-ischemic causes of SCD. The classification to ischemic subcategory included the presence of coronary artery pathology defined by the presence of at least one of the following criteria: an acute intracoronary thrombus, plaque rupture or erosion, hemorrhage within a plaque, a critical coronary stenosis exceeding 75% in a major coronary artery, or if the coronary stenosis ranged from 50% to 75%, and there was concomitant coronary artery disease related myocardial disease, such as an infarction scar, with no other identified cause of SCD. The cases that did not meet the criteria for ischemic SCD were classified as non-ischemic, including various types of cardiomyopathies. The details of the FINGESTURE study protocol have been published previously [7,8]. The study was approved by the Ethics Committee of Northern Ostrobothnia Hospital District and complies with the Declaration of Helsinki. Permissions to gather data from the documents of medico-legal cause-of-death investigation were obtained from the Finnish Institute for Health and Welfare and from the Regional State Administrative Agency of Northern Finland.

### 2.1 Statistical analyses

The χ^2^ test was used to assess the statistical significance of the differences of the distributions between the study groups of interest. The Kruskal-Wallis test was used to evaluate the statistical significance of the difference in the incidence of SCD between the days from Monday to Thursday versus the days from Friday to Sunday. The continuous variables are shown as mean ± standard deviation. IBM SPSS Statistic Version 26 was used for the statistical analyses. The p-values <0.05 were considered statistically significant.

## 3 Results

The characteristics of the unexpected SCD victims of the present study are shown in Table 1. The mean age of the SCD victims was 64.9 ± 12.4 years (age range 99 years). The majority of the SCD victims were males (n = 4,631, 78.9%). Autopsy data based heart weight, height, weight and body mass index of the SCD victims are shown separately for males and females in Table 1. In a majority of cases the cause of SCD was ischemic (n = 4,392, 74.8%), and in a minority of cases non-ischemic (n = 1,477, 25.2%). The medical history is also shown in Table 1. However, the data are somewhat incomplete and most probably underestimates the occurrence of diseases among these subjects.

**Table 1:** Characteristics of the unexpected sudden cardiac death victims.

Variable	SCD victims
	n = 5869
Age (years)	64.9 ± 12.4
Males	4631 (78.9%)
Heart weight (grams)	
males	500.8 ± 127.5
females	411.8 ± 104.4
Height (centimeters)	
males	173.3 ± 7.9
females	159.6 ± 7.6
Weight (kilograms)	
males	83.0 ± 21.4
females	72.3 ± 22.3
Body mass index (kg/m^2^)	
males	27.5 ± 6.0
females	28.3 ± 7.6
Cause of SCD:	
Ischemic	4392 (74.8%)
Non-ischemic	1477 (25.2%)
Medical history	
CAD	18.5%
Diabetes	19.5%
Heart Failure	8.9%
Hypertension	5.3%
Dyslipidemia	12.4%
Acute myocardial infarction	7.0%
Angina pectoris	7.2%
Dyspnea	3.7%

The values are mean ± SD or the number of sudden cardiac death (SCD) victims (percentages). CAD = coronary artery disease.

**Fig. 1:**
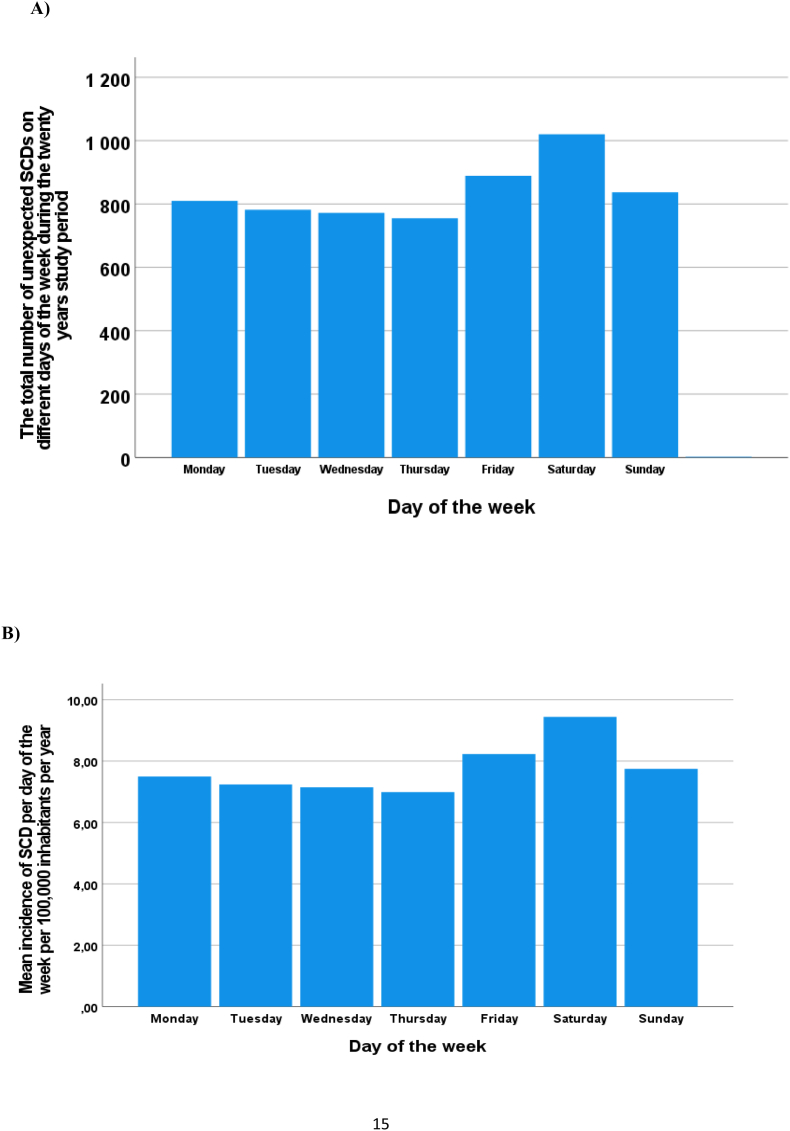
The total number of unexpected sudden cardiac deaths (SCDs) on different days of the week during the twenty years of follow-up from 1998 to 2017 in the northern Finland region with on average 600,000 inhabitants (**A**). Mean incidence of unexpected SCD on different days of the week per 100,000 inhabitants per year during the twenty-year follow-up period. The mean incidence of unexpected SCD was higher during the days from Friday to Sunday (peaking on Saturday) than during the days from Monday to Thursday (8.54 ± 0.72 vs. 7.22 ± 0.19 SCDs per day of the week per 100,000 inhabitants per year, p < 0.001) (**B**).

During the twenty years of follow-up time, the number of unexpected SCD victims was higher during the days from Friday to Sunday (peaking on Saturday) than during the days from Monday to Thursday (Fig. 1A). The mean incidence of unexpected SCD was higher during the days from Friday to Sunday (with peak incidence on Saturday) than during the days from Monday to Thursday (8.54 ± 0.72 vs. 7.22 ± 0.19 SCDs per day of the week per 100,000 inhabitants per year, p < 0.001) (Fig. 1B).

**Fig. 2:**
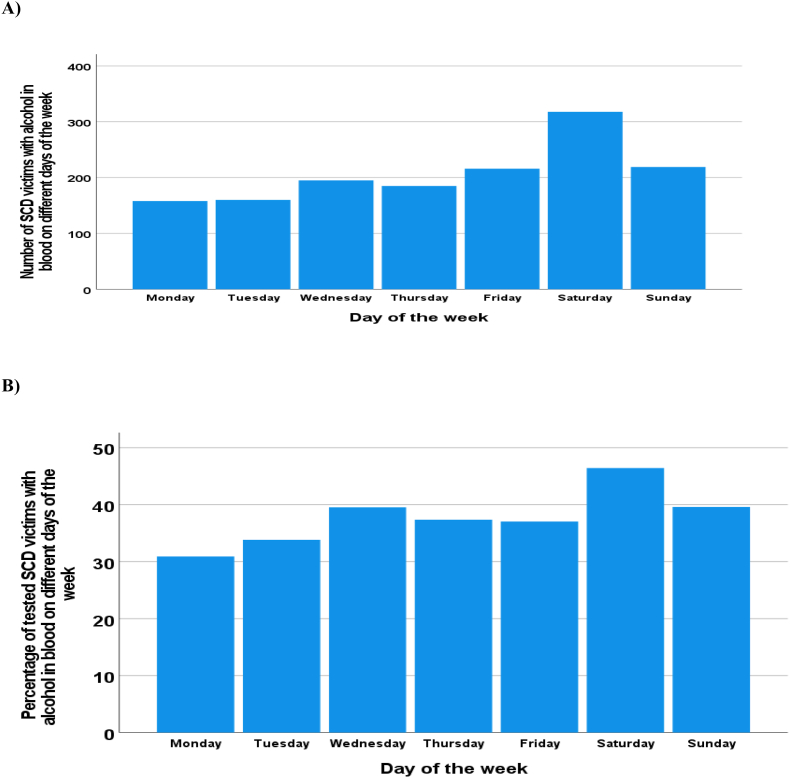
The number of sudden cardiac death (SCD) victims with alcohol in blood on different days of the week during the twenty years of follow-up (**A**). The percentage of the tested SCD victims with alcohol in blood on different days of the week (**B**). Blood alcohol levels were measured in 3795 SCD victims, i.e., whenever there was any suspicion of alcohol use prior to SCD. The blood alcohol level was tested in 63.1%, 60.5%, 63.9%, 65.6%, 65.6%, 67.2% and 66.1% of the cases who experienced SCD on Monday, Tuesday, Wednesday, Thursday, Friday, Saturday, or Sunday, respectively.

The number of SCD victims with alcohol in blood was higher at weekends (peaking on Saturday) (Fig. 2A). The proportion of the tested SCD victims with alcohol in blood was highest on Saturdays (Fig. 2B). In SCD victims with alcohol in blood, the blood alcohol concentration was statistically significantly higher in those who succumbed at weekends (during the days from Friday to Sunday), (1.36 ± 0.84 ‰ versus 1.26 ± 0.86 ‰, p = 0.027).

**Fig. 3:**
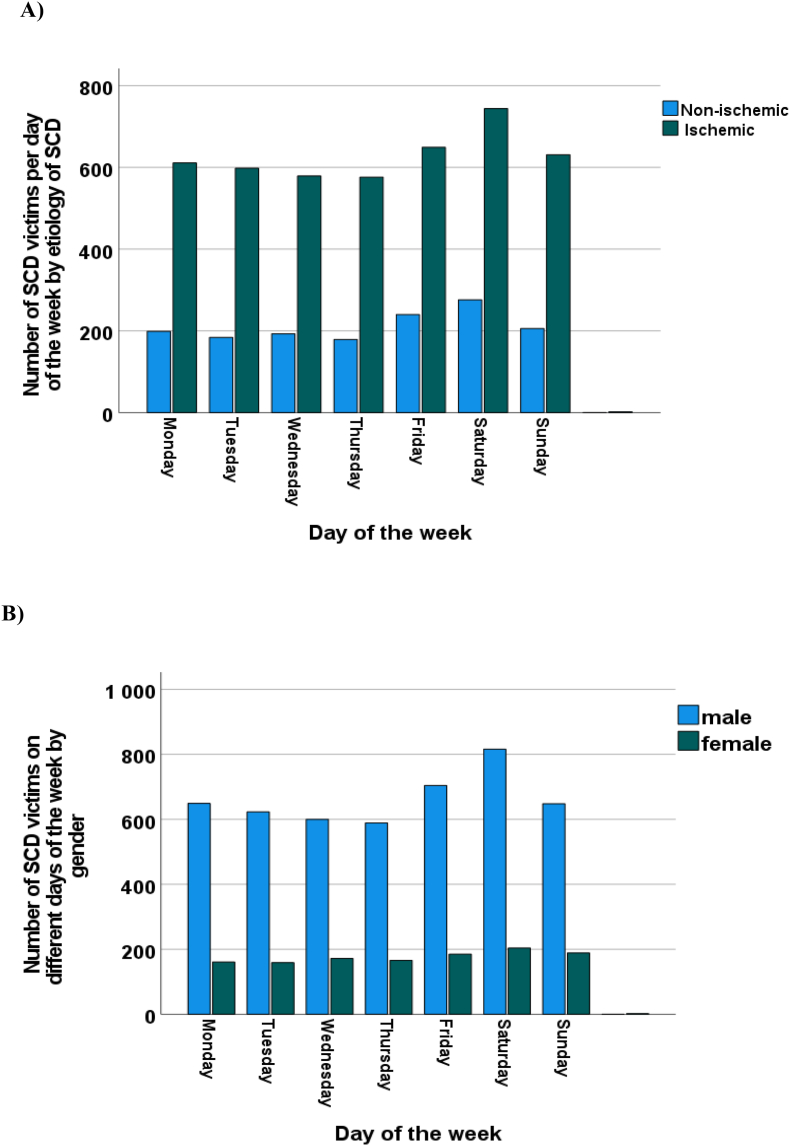
Number of unexpected sudden cardiac death (SCD) victims on different days of the week by the etiology of SCD during the twenty years of follow-up (**A**). Number of SCD victims on different days of the week by sex during the twenty years of follow-up (**B**).

Regardless of the etiology of SCD (ischemic or non-ischemic), the distribution of the number of SCDs was similar between the days of the week compared with the whole SCD cohort, and the distribution of the number of SCDs did not differ statistically significantly between the ischemic and non-ischemic cases (p = 0.49) (Fig. 3A). The day-of-the-week distributions of unexpected SCD showed no statistically significant difference between sexes (p = 0.12) (Fig. 3B). The distribution of the number of both male and female SCD victims between the days of the week was similar when compared with the whole SCD cohort.

## 4 Discussion

In the present study with a prospectively collected large consecutive series of unexpected SCD victims who underwent a medico-legal autopsy, we found that the occurrence of SCD was more common on the days from Friday to Sunday (with a peak on Saturday) than on the days from Monday to Thursday. The distribution of the SCD victims with alcohol in blood had a similar pattern during the days of the week as the distribution of the number of SCD victims, and the blood alcohol concentration was higher at weekends. Regardless of sex or ischemic versus non-ischemic etiology of SCD, the distribution of the occurrence of SCD among the days of the week was similar compared with the whole SCD cohort.

There are also previous studies which have evaluated the day-of-the-week (septadian) variations in the incidence of sudden death. Some studies have found higher incidence of SCD on Mondays. Arntz et al. analyzed the emergency medical system data of 24,061 patients with sudden death of presumed cardiac origin in Berlin and found peaks in the occurrence during morning hours on Mondays [3]. Peters et al. evaluated the septadian distribution of life-threatening ventricular arrhythmias in 683 consecutive patients with implantable cardioverter-defibrillators (ICDs). They found a prominent peak on Mondays, a midweek decline, a secondary peak from Thursday to Friday, and a nadir from Saturday to Sunday. The pattern was not observed in patients receiving beta blockers [9]. In another study, Peters et al. concluded that the physiologic modulators of circadian and septadian rhythms might be different [10]. Peckova et al. evaluated 5284 cases of sudden death from the Seattle Fire Department and found a higher incidence of sudden death on Mondays and a trend for a decreasing occurrence of cardiac arrests from Mondays to weekend [4]. In contrast, in our present study where all age ranges were included and non-cardiac sudden deaths were carefully excluded based on meticulous examinations in medico-legal autopsies, the incidence of unexpected SCD was higher during the days from Friday to Sunday, peaking on Saturday, regardless of sex or ischemic versus non-ischemic etiology of SCD. In a Chinese population, the septadian distribution of the frequency of acute myocardial infarction occurrence was similar with the septadian distribution of SCD occurrence in our present study [11].

Based on a few newer studies, it has been speculated that there might recently have been a loss of circadian and septadian variation in sudden cardiac arrest [6]. In a post-hoc analysis of the Sudden Cardiac Death in Heart Failure trial (SCD-HeFT) with a relatively small number of patients, no expected circadian or septadian patterns of ICD therapies were found in general, although in patients not on beta blockers a significant Monday peak in ICD therapies was observed [12]. It is noteworthy that the SCD-HeFT patients were high-risk patients with very low left ventricular ejection fraction. It may be that the loss of septadian variation of ICD therapies in these high-risk patients does not represent septadian variation of SCDs which occur numerically more commonly in low-risk patients and general population. In the analysis of the Oregon Sudden Unexpected Death study, the researchers identified 1535 patients who presented with witnessed sudden cardiac arrest during the years 2002–2014 and were attended by the emergency medical services. They found no peak in sudden cardiac arrest on Mondays. Only a minority of those who deceased underwent an autopsy and detailed information of the cause of sudden cardiac arrest was limited [5]. It is also noteworthy that in general, 70–80% of the SCD victims have clinical or subclinical ischemic heart disease and that one in three patients with an acute ischemic event dies suddenly before getting to a hospital. In our present study with a series of 5869 prospectively collected consecutive unexpected SCD victims, all of whom underwent medico-legal autopsy during the years 1998–2017, the day-of-the-week variation of SCD was clear.

The explanation for our finding of higher incidence of unexpected SCD at weekends with a peak on Saturdays remains partly enigmatic. Hypothetically, staying awake late and a possible increase in alcohol intake at weekends may worsen the quality of sleep. Alcohol intake may also increase blood pressure, cause hyper-adrenergic state, electrolyte disturbances, repolarization disturbances, and worsening of sleep apnea and ischemia, which may increase the risk for acute coronary syndromes, life-threatening ventricular arrhythmias and SCD in vulnerable subjects [13]. It is plausible to speculate, partly based on our present findings, that the septadian pattern of alcohol use may have contributed to the day-of-the-week distribution of SCD with a peak on Saturdays. In line with our findings, a study in a Lithuanian population showed a higher number of deaths from cardiovascular diseases on Saturdays, Sundays, as well as Mondays, possibly with binge drinking as a contributing factor [14]. Another hypothetical explanation for our present findings could be that physical activity may trigger SCD [15]. Generally, people are quite active during the weekends, and various activities and physical exercise during the weekend may have contributed to the risk of SCD, especially in previously inactive and unfit people.

Our study has some limitations. We found a similar distribution of the number of SCD victims with alcohol in blood and the occurrence of SCD among the days of the week, but this does not prove causality. Furthermore, blood alcohol level was measured whenever there was any suspicion of alcohol use prior to SCD, but it was not measured in all SCD victims. We had incomplete data about physical activity before SCD, and could not explore whether there was a difference between the weekend and weekdays in that matter. Therefore, the discussion about the causes of the finding of higher incidence of unexpected SCD at weekends with a peak on Saturdays remains speculative.

In conclusion, the present analysis of the large series of consecutive victims of unexpected SCD showed that SCDs occurred more commonly during the days from Friday to Sunday with a peak on Saturday regardless of sex or ischemic versus non-ischemic etiology of SCD.

## Funding

The study was supported by a grant from 10.13039/501100014438Business Finland.

## CRediT authorship contribution statement

**Hanna Latola:** Conceptualization, Formal analysis, Investigation, Methodology, Writing – original draft. **Severi M. Mattila:** Conceptualization, Investigation, Methodology, Writing – review & editing. **Lasse Pakanen:** Conceptualization, Investigation, Methodology, Resources, Writing – review & editing. **Lauri Holmström:** Conceptualization, Investigation, Methodology. **Janna Kauppila:** Conceptualization, Investigation, Methodology. **Olavi H. Ukkola:** Conceptualization, Investigation, Methodology. **M Juhani Junttila:** Conceptualization, Investigation, Methodology. **Heikki V. Huikuri:** Conceptualization, Investigation, Methodology. **Juha S. Perkiömäki:** Conceptualization, Formal analysis, Investigation, Methodology, Project administration, Resources, Supervision, Writing – original draft.

## Declaration of competing interest

None declared.

## References

[1] Lynge T.H., Risgaard B., Banner J., Nielsen J.L., Jespersen T., Stampe N.K. (2021). Nationwide burden of sudden cardiac death: a study of 54,028 deaths in Denmark. Heart Rhythm.

[2] Arntz H.R., Müller-Nordhorn J., Willich S.N. (2001). Cold Monday mornings prove dangerous: epidemiology of sudden cardiac death. Curr. Opin. Crit. Care.

[3] Arntz H.R., Willich S.N., Schreiber C., Brüggemann T., Stern R., Schultheiss H.P. (2000). Diurnal, weekly and seasonal variation of sudden death. Population-based analysis of 24,061 consecutive cases. Eur. Heart J..

[4] Peckova M., Fahrenbruch C.E., Cobb L.A., Hallstrom A.P. (1999). Weekly and seasonal variation in the incidence of cardiac arrests. Am. Heart J..

[5] Ni Y.M., Rusinaru C., Reinier K., Uy-Evanado A., Chugh H., Stecker E.C. (2019). Unexpected shift in circadian and septadian variation of sudden cardiac arrest: the Oregon Sudden Unexpected Death Study. Heart Rhythm.

[6] Ramireddy A., Chugh S.S. (2021). Do peak times exist for sudden cardiac arrest?. Trends Cardiovasc. Med..

[7] Haukilahti M.A.E., Holmström L., Vähätalo J., Kenttä T., Tikkanen J., Pakanen L. (2019). Sudden cardiac death in women. Circulation.

[8] Holmström L., Juntunen S., Vähätalo J., Pakanen L., Kaikkonen K., Haukilahti A. (2022). Plaque histology and myocardial disease in sudden coronary death: the Fingesture study. Eur. Heart J..

[9] Peters R.W., McQuillan S., Resnick S.K., Gold M.R. (1996). Increased Monday incidence of life-threatening ventricular arrhythmias. Experience with a third-generation implantable defibrillator. Circulation.

[10] Peters R.W., McQuillan S., Gold M.R. (1999). Interaction of septadian and circadian rhythms in life-threatening ventricular arrhythmias in patients with implantable cardioverter-defibrillators. Am. J. Cardiol..

[11] Zhou R.H., Xi B., Gao H.Q., Liu X.Q., Li Y.S., Cao K.J. (1998). Circadian and septadian variation in the occurrence of acute myocardial infarction in a Chinese population. Jpn. Circ. J..

[12] Patton K.K., Hellkamp A.S., Lee K.L., Mark D.B., Johnson G.W., Anderson J. (2014). Unexpected deviation in circadian variation of ventricular arrhythmias: the SCD-HeFT (sudden cardiac death in heart failure trial). J. Am. Coll. Cardiol..

[13] Perkiömäki J., Hookana E., Kaikkonen K., Junttila J., Kortelainen M.L., Huikuri H. (2016). Blood alcohol in victims of sudden cardiac death in northern Finland. Europace.

[14] Chenet L., Britton A., Kalediene R., Petrauskiene J. (2001). Daily variations in deaths in Lithuania: the possible contribution of binge drinking. Int. J. Epidemiol..

[15] Toukola T., Hookana E., Junttila J., Kaikkonen K., Tikkanen J., Perkiömäki J., Kortelainen M.L., Huikuri H.V. (2015). Sudden cardiac death during physical exercise: characteristics of victims and autopsy findings. Ann. Med..

